# Prostatic ischemia induces ventral prostatic hyperplasia in the SHR; possible mechanism of development of BPH

**DOI:** 10.1038/srep03822

**Published:** 2014-01-22

**Authors:** Motoaki Saito, Panagiota Tsounapi, Ryo Oikawa, Shogo Shimizu, Masashi Honda, Takehiro Sejima, Yukako Kinoshita, Shuhei Tomita

**Affiliations:** 1 Department of Pharmacology, Kochi University School of Medicine, Nankoku, 783-8505, Japan; 2 Division of Urology, Tottori University School of Medicine, 36-1 Nishi-cho, Yonago, 683-8503, Japan; 3 Division of Molecular Pharmacology, Tottori University School of Medicine, 86 Nishi-cho, Yonago, 683-8503, Japan

## Abstract

In the light of increasing evidence that benign prostatic hyperplasia is associated with cardiovascular disease, we have investigated the relationship between prostatic blood flow and prostatic hyperplasia in the spontaneously-hypertensive-rat (SHR). Twelve-week-old male SHRs were treated with nicorandil for six weeks. Wistar-Kyoto rats were used as controls. Six weeks after nicorandil treatment, blood pressure and the prostatic blood flow were estimated, and tissue levels of malondialdehyde, HIF-1α, TGF-β1, bFGF, dihydrotestosterone, and α-SMA were measured. SHRs showed significant increases in blood pressure, tissue levels of malondialdehyde, HIF-1α, TGF-β1, bFGF, α-SMA and a significant decrease in the prostatic blood flow. Although treatment with nicorandil failed to alter the blood-pressure and α-SMA, it significantly ameliorated the increased levels of malondialdehyde, HIF-1α, TGF-β1, and bFGF. There were no significant differences in tissue levels of dihydrotestosterone among any groups. These data indicate that development of prostatic hyperplasia may be associated with prostatic hypoxia, which nicorandil prevents via its effect to increase the blood flow.

Benign prostatic hyperplasia (BPH) and benign prostatic enlargement (BPE) are common age-associated disorders experienced by men worldwide, and the conditions usually become clinically apparent after the age of 40 years old. The clinical relevance of BPH/BPE is underscored by the fact that up to 50% of elderly men develop lower urinary tract symptoms due to BPH/BPE, and that trans urethral resection of the prostate (TURP) remains the gold-standard therapy against BPH, with a lifetime risk for surgery of around 25–30%^1^,^2^. BPH/BPE originates in the transition zone of the prostate and consists of a nodular overgrowth of the epithelium and fibromuscular tissue within transitional zone and periurethral areas^1^,^2^. Although the pathogenesis of BPH/BPE is poorly understood and thought to be multifactorial, prostate tissue-remodeling in the transition zone is characterized by: (i) hypertrophic basal cells, (ii) altered secretions of luminal cells leading to calcification, clogged ducts and inflammation, (iii) lymphocytic infiltration with production of proinflammatory cytokines, (iv) increased radical oxygen species (ROS) production that damages epithelial and stromal cells, (v) increased basic fibroblast growth factor (bFGF) and transforming growth factor beta 1 (TGF-β1) production leading to stromal proliferation, transdifferentiation and extracellular matrix production, (vi) altered autonomous innervation that decreases relaxation and leads to a high adrenergic tonus, and (vii) altered neuroendocrine cell function and release of neuroendocrine peptides^2^. In many mammalians studies, only the chimpanzee and dog have been known to develop BPH/BPE except for man^3^,^4^.

Spontaneously hypertensive rats (SHRs), a commonly used model of genetic hypertension, have been found to exhibit hyperplastic morphological abnormalities in the ventral prostate^5^,^6^,^7^. Yamashita and his associates reported that the hyperplastic changes in the ventral prostate in the SHR are developed with advancing age in the SHR, and that hyperplastic changes in the ventral prostate are observed as early as 15 weeks of age^8^. These reports indicate that the prostate, especially the ventral prostate of the SHR can be a good model for human BPH/BPE. In addition, this rodent model is inexpensive and quite easy to handle compared with the chimpanzee or dog. Yono and his associates reported that blood flow in the dorsolateral and ventral prostate in the SHR is significantly decreased compared to that in the Wistar-Kyoto rats (WKYs), and that chronic treatment with an α1-blocker, doxazosin normalizes this decreased prostatic blood flow^9^. Additionally, the hyperplastic changes in the SHR prostate were inhibited by chronic administration of another α1-blocker, terazosin^10^. These data suggest that prostatic blood flow plays an important role in the development of prostatic hyperplasia in the SHR.

Oxidative stress has been related to the etiopathogenesis of several chronic diseases and plays a paramount role in the aging proces^11^. Of the many biological targets of oxidative stress, lipids are the most involved class of biomolecules. Lipid oxidation gives rise to a number of secondary products. These products are mainly aldehydes, with the ability to exacerbate oxidative damage^12^. Longevity and high reactivity allow these molecules to act inside and outside the cells, interacting with biomolecules such as nucleic acids and proteins, often irreversibly damaging the delicate mechanisms involved in cell functionality. The direct measurement of ROS is very difficult due to their very short half life. Alternatively, several products induced by lipid peroxidation can be used as precise indicator of lipid damage. Malondialdehyde (MDA) is the principal and most studied product of polyunsaturated fatty acid peroxidation.

Nicorandil (2-nicotiamidoethyl-nitrate ester) is worldwidely used in the pharmacotherapy against angina and acute heart failure^13^. As nicorandil is a hybrid compound that consists of an *N*-(2-hydroxyethyl) nicotinamide vitamin group and an organic nitrate moiety, nicorandil is thought to have dual actions, a nitrate-like action and an opening of K_ATP_ channels action^14^. In canine arterial muscle, these K_ATP_ channel openers reduce the force of contraction exclusively by opening K_ATP_ channels, because their negative inotropic effects are totally blocked by the quaternary ammonium K_ATP_ channel blockers, tetraethylammonium or tetrabutylammonium^13^,^14^. Recently, we reported that nicorandil increases the bladder blood flow in the SHR, and that chronic treatment with nicorandil ameliorates hypertension-related bladder dysfunction in the SHR^15^.

From these reports, one hypothesis that can be suggested is that mild hypoxia in the prostate induces up-regulation of hypoxia-inducible factor 1 (HIF-1), the master regulator of oxygen homeostasis, and ROS, which subsequently activate TGF-β1 and bFGF in the prostate, leading to stromal proliferation, transdifferentiation and extracellular matrix production. This is one of the possible mechanisms in the development of BPH/BPE. If this hypothesis is true, normalization of the prostatic blood flow should inhibit the development of the prostatic hyperplasia. In the present study, we have attempted to investigate the effect of chronic administration of nicorandil on the prostatic blood flow and hyperplasia in the SHR.

## Results

### General features of the experimental animals

Table 1 shows the general features of the experimental rats. The body weight and prostate weight in the SHR were significantly smaller than those in the WKY. However, prostate-body weight ratio (PBR) in the SHR was significantly higher than that in the WKY. Although treatment with nicorandil failed to increase the body weight in the SHR, the PBR in the Nic10 group was not significantly different compared either to the SHR or to the WKY groups (p = 0.92 or p = 0.09, respectively). Even though the heart rate in the SHR was similar to the WKY, blood pressure (systolic, mean and diastolic) in the SHR was significantly higher than that in the WKY. Any dose of nicorandil treatment failed to reduce the blood pressure in the SHR.

### Nicorandil could restore the decreased blood flow of the prostate observed in the SHR

**Figure 1:**
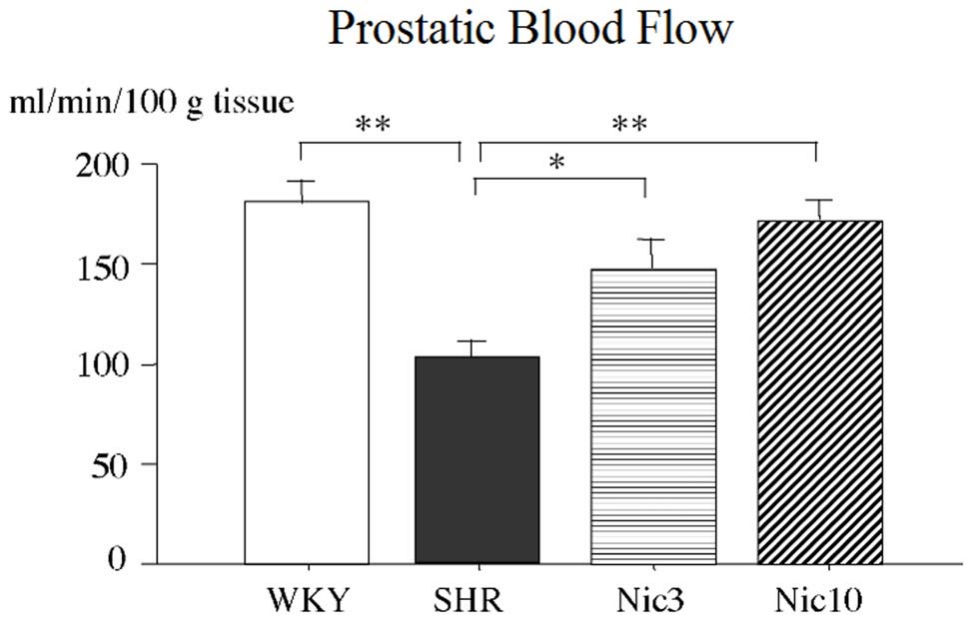
Blood flow in the prostate. WKY: 18-week-old Wistar-Kyoto rat group; SHR: 18-week-old SHR group; Nic3: 18-week-old SHRs treated with nicorandil at a daily dose of 3 mg/kg, i.p.; Nic10: 18-week-old SHRs treated with nicorandil at a daily dose of 10 mg/kg, i.p.; *: Significantly different between groups (P < 0.05 is level of significance); **: Significantly different between groups (P < 0.01 is level of significance).

Previous study has demonstrated a decreased blood flow in the prostate of the SHR model compared to the WKY control^9^ indicating this defect as one of the causes of BPH in the SHR. We, therefore, examined the prostatic blood flow by the hydrogen clearance method. Our results revealed a significantly lower prostatic blood flow in the SHR group compared to the WKY group (57% of the WKY). Treatment with the low dose of nicorandil significantly recovered the prostatic blood flow, whereas the high dose of nicorandil significantly recovered the prostatic blood flow to the level of the WKY (Figure 1).

### The SHR prostate showed significantly increased levels of oxidative stress

**Figure 2:**
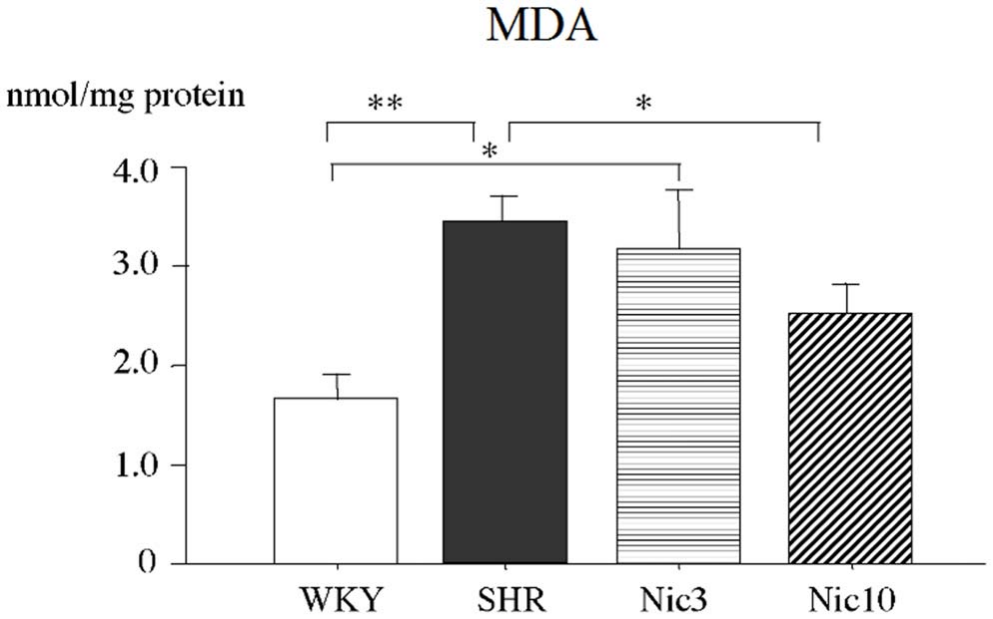
Tissue levels of MDA in the prostate. WKY: 18-week-old Wistar-Kyoto rat group; SHR: 18-week-old SHR group; Nic3: 18-week-old SHRs treated with nicorandil at a daily dose of 3 mg/kg, i.p.; Nic10: 18-week-old SHRs treated with nicorandil at a daily dose of 10 mg/kg, i.p.; *: Significantly different between groups (P < 0.05 is level of significance); **: Significantly different between groups (P < 0.01 is level of significance).

Next we examined the levels of oxidative stress in the prostate. Since ROS are very difficult to detect, we focused on MDA which is a marker of lipid peroxidation. The tissue levels of MDA in the experimental rats are shown in the Figure 2. The SHR demonstrated significantly increased tissue levels of MDA compared to the WKY. Although the tissue levels of MDA in the Nic3 group were similar to the SHR group, those in the Nic10 group were significantly smaller than the respective in the SHR group.

### Up-regulation of the tissue levels of HIF-1, TGF-β1, bFGF and dihydrotestosterone (DHT) in the prostate of the SHR

**Figure 3:**
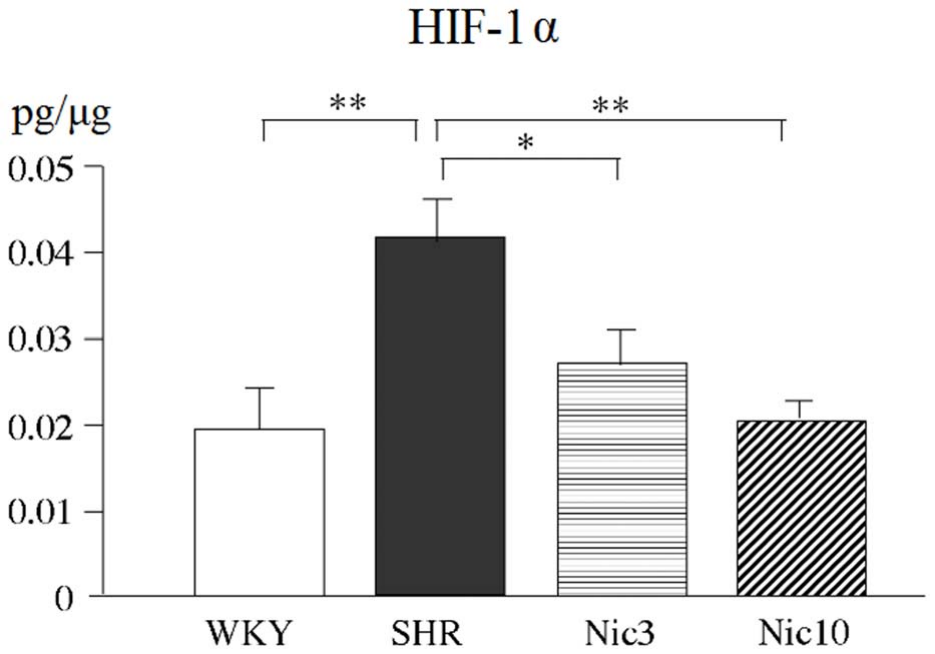
Tissue levels of HIF-1α in the prostate. WKY: 18-week-old Wistar-Kyoto rat group; SHR: 18-week-old SHR group; Nic3: 18-week-old SHRs treated with nicorandil at a daily dose of 3 mg/kg, i.p.; Nic10: 18-week-old SHRs treated with nicorandil at a daily dose of 10 mg/kg, i.p.; *: Significantly different between groups (P < 0.05 is level of significance); **: Significantly different between groups (P < 0.01 is level of significance).

HIF-1 is a heterodimeric transcription factor containing an inducible expressed HIF-1α subunit and a constititutively expressed HIF-1β subunit. Under hypoxic conditions, the HIF-1α subunit accumulates. Considering that probably a mild hypoxia would be induced by the impaired prostatic blood flow, which we observed in the SHR, we evaluated the HIF-1α levels in the prostate. The tissue levels of HIF-1α in the SHR group were significantly higher than those in the WKY group indicating the hypoxic condition of the prostate in the SHR. Treatment with both doses of nicorandil significantly decreased the tissue levels of HIF-1α in the SHR. The tissue levels of HIF-1α in the Nic10 were similar to those in the WKY group (Figure 3).

**Figure 4:**
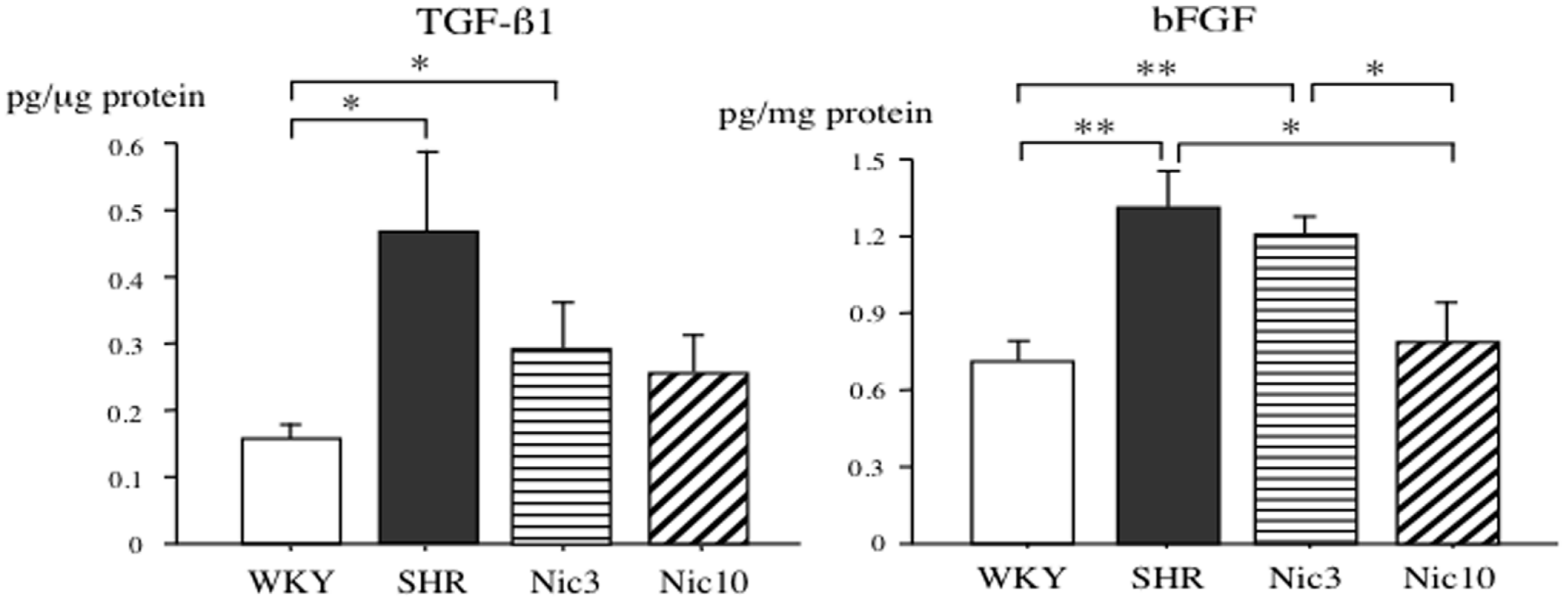
Tissue levels of TGF-β1 and bFGF in the prostate. Right panel: tissue levels of TGF-β1 in the prostate; Left panel: tissue levels of bFGF in the prostate. WKY: 18-week-old Wistar-Kyoto rat group; SHR: 18-week-old SHR group; Nic3: 18-week-old SHRs treated with nicorandil at a daily dose of 3 mg/kg, i.p.; Nic10: 18-week-old SHRs treated with nicorandil at a daily dose of 10 mg/kg, i.p.; *: Significantly different between groups (P < 0.05 is level of significance); **: Significantly different between groups (P < 0.01 is level of significance).

Previously TGF-β and bFGF have been suggested to have important roles in the regulation of stromal cells homeostasis^16^. We have, therefore, examined the tissue levels of TGF-β1 and bFGF in the prostate. The tissue levels of TGF-β1 in the SHR group were significantly higher than those in the WKY group. The tissue levels of TGF-β1 in the Nic3 group did not statically decrease compared to the SHR group and were significantly higher compared to the WKY. The Nic10 group demonstrated tissue levels of TGF-β1 partially normalized as there was not any statistically significant difference either compared to the SHR or WKY groups. The tissue levels of bFGF in the SHR group were significantly higher than those in the WKY group. Although the low dose of nicorandil statically failed to improve the tissue levels of bFGF in the Nic3 group compared to the SHR group, the high dose of nicorandil in the Nic10 group returned the bFGF tissue levels back to the same as the respective in the WKY group. Additionally bFGF tissue levels in Nic10 group were statically significantly different from the SHR or Nic3 groups (Figure 4).

**Figure 5:**
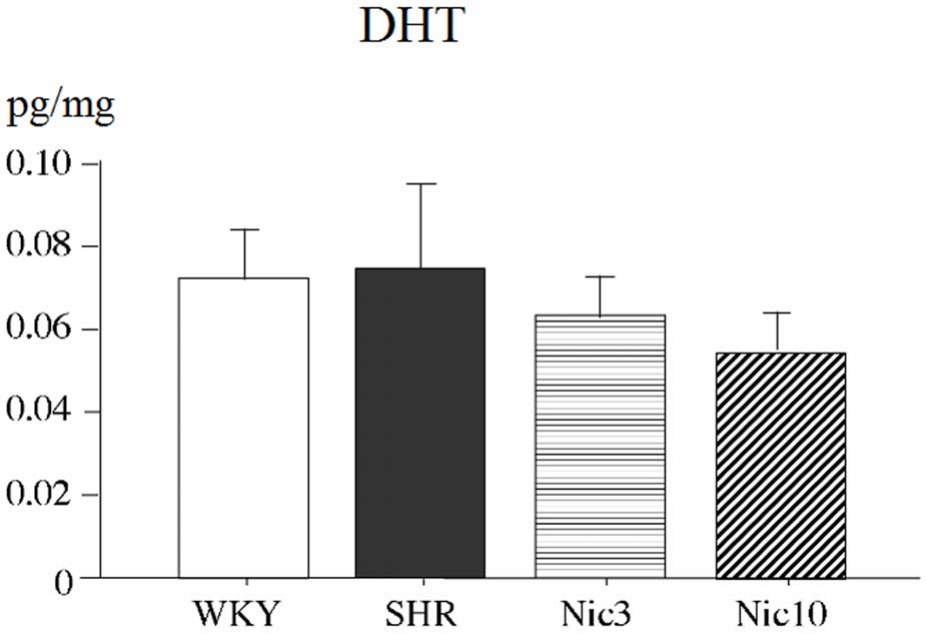
Tissue levels of DHT in the prostate. WKY: 18-week-old Wistar-Kyoto rat group; SHR: 18-week-old SHR group; Nic3: 18-week-old SHRs treated with nicorandil at a daily dose of 3 mg/kg, i.p.; Nic10: 18-week-old SHRs treated with nicorandil at a daily dose of 10 mg/kg, i.p.

The whole prostate gland is an androgen-sensitive organ, with both the epithelium and stroma under the control of androgen. In order to confirm the possible effect of androgen, we measured the tissue levels of DHT in the prostate, and the data are shown in Figure 5. The tissue levels of DHT in the SHR group were similar to those in the WKY group. In addition both doses of treatment with nicorandil did not alter tissue levels of DHT in the SHR prostate. In this case, the participation of androgens is excluded as a possible mechanism of the pathogenesis of the BPH.

### Increased expression of the alpha-Smooth muscle actin (α-SMA) protein in the SHR prostate

**Figure 6:**
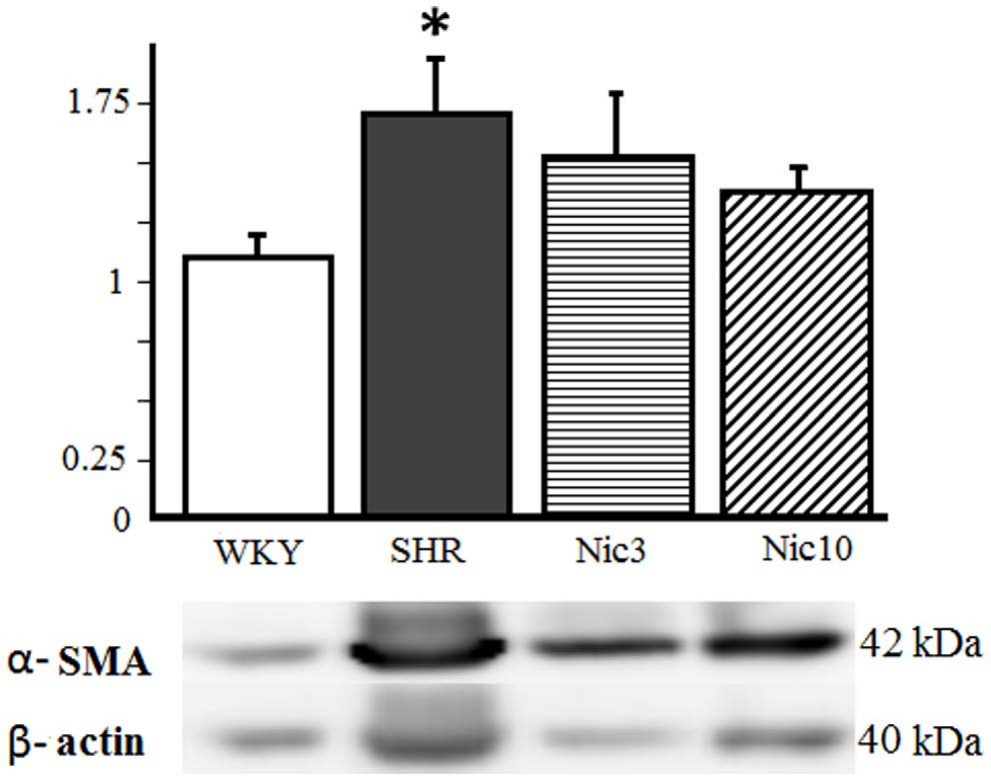
α-SMA protein expression in the prostate. *α*-SMA: alpha-Smooth muscle actin; WKY: 18-week-old Wistar-Kyoto rat group; SHR: 18-week-old SHR group; Nic3: 18-week-old SHRs treated with nicorandil at a daily dose of 3 mg/kg, i.p.; Nic10: 18-week-old SHRs treated with nicorandil at a daily dose of 10 mg/kg, i.p.; *: Significantly different between groups (P < 0.05 is level of significance).

The expression of α-SMA is used as a marker for prostatic fibroblasts within the prostatic stroma. Therefore we examined the α-SMA protein expression in the prostatic tissue by Western blot. We found a significant increase in the α-SMA protein expression in the SHR group compared to the WKY group. The treatment with nicorandil slightly decreased the α-SMA protein expression in a dose dependent manner, but it was not statistically different compared to the SHR group; neither was significantly different compared to the WKY group (Figure 6).

### The SHR presented changes in the histopathology of the prostatic tissue

**Figure 7:**
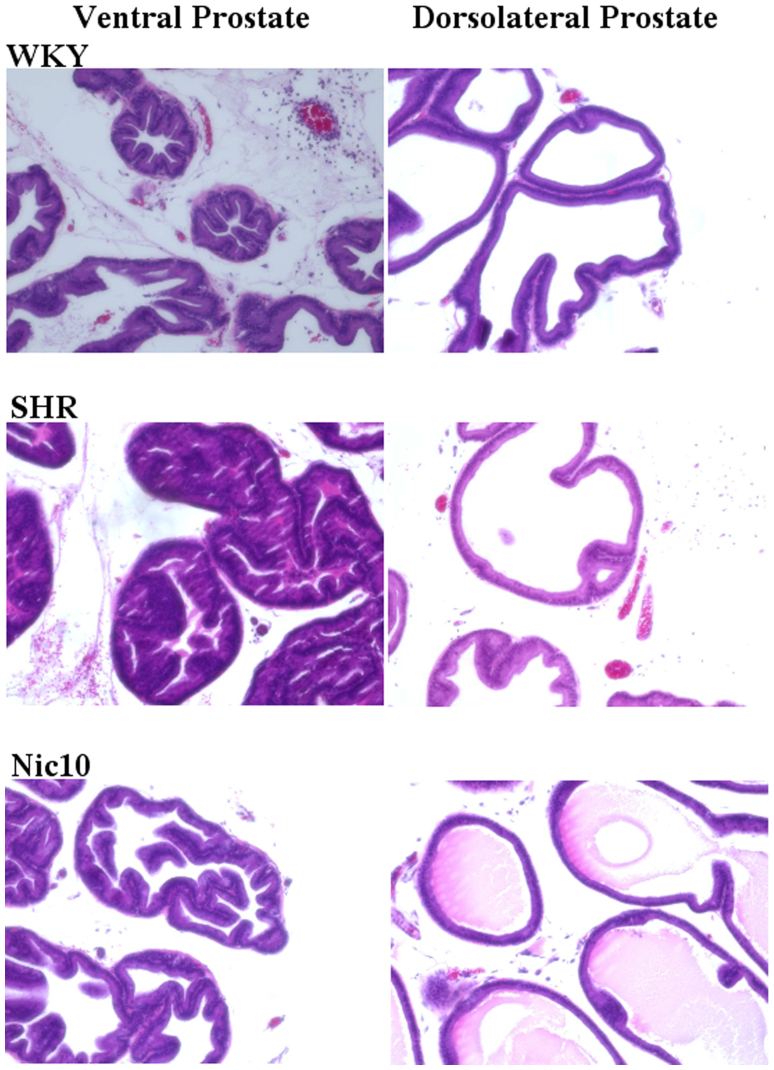
Typical histological changes in the ventral and dorsolateral prostate. WKY: 18-week-old Wistar-Kyoto rat group; SHR: 18-week-old SHR group; Nic10: 18-week-old SHRs treated with nicorandil at a daily dose of 10 mg/kg, i.p. Original magnification ×200.

Hematoxylin-eosin (H&E) staining slides were prepared in order to observe the histopathological changes of the ventral and dorsolateral prostate. In figure 7 H&E stains from the WKY, SHR and Nic10 groups (×200) are presented. The ventral prostate in the WKY group showed regular, unfolded closely packed acini tapered by low cuboidal cells showing a uniform monolayered arrangement. In contrast to the WKY group, the ventral prostate in the SHR group showed epithelial cells being taller in the shape with irregularities in the nuclear arrangement. Treatment with nicorandil normalized these abnormalities. However, significant alterations in the dorsolateral prostate were not found either in the SHR group or the Nic10 group.

## Discussion

The aim of the present study was to investigate whether or not our initial hypothesis that mild hypoxia in the prostate through a cascade of events is a possible mechanism, which contributes to the development of BPH/BPE. Additionally whether the chronic treatment with nicorandil could restore the blood flow and inhibit the development of the prostatic hyperplasia. The SHR model which we used in our study, demonstrated significantly higher blood pressure, both systolic and diastolic compared to the WKY group. The SHR exhibited also a hyperplastic ventral prostate as demonstrated by the significantly higher PBR compared to the WKY group. Furthermore the SHR group showed significantly decreased prostatic blood flow, and significantly increased levels of MDA, HIF-1α, TGF-β1, bFGF and α-SMA in the prostate. The data we obtained provide support to a possible mechanism of the development of the prostatic hyperplasia. This mild hypoxia subsequently induces up-regulation of oxidative stress and HIF-1α levels, and consequentially these factors induce an increase of TGF-β1 and bFGF in the prostate, in a direct or indirect way. Although prostate is an androgen dependent organ and also androgens are established risk factors for the development of BPH/BPE, we measured the prostate tissue levels of DHT. In the present study there was no difference in the DHT tissue levels in the prostate among any of the groups. This is an additional evidence, which supports our hypothesis; this means that the development of the BPH in our SHR model was mainly induced by the decreased prostatic blood flow, and furthermore by the mild hypoxia and not controlled by the androgen levels. The six-week-treatment with the high dose of nicorandil could significantly increase the blood flow and significantly decrease the MDA, HIF-1α, and bFGF levels; while it partially normalized the TGF-β1 and α-SMA levels in the prostate.

Ghafar et al. proposed that the prostatic vascular system might play a role in the development of BPH^17^. In addition, Berger et al. reported that atherosclerosis is a risk factor for BPH^18^. Furthermore, Berger et al. reported that an age-related impairment of blood supply to the lower urinary tract has a role in the development of BPH, and that vascular damage might cause chronic ischemia and thus be a contributing factor in the pathogenesis of BPH^18^,^19^. These clinical and translational studies encourage and support our hypothesis that mild hypoxia induced by the impaired blood flow in the prostate induces development of prostatic hyperplasia.

ROS can activate TGF-β1 either directly or indirectly via the activation of proteases, and in addition TGF-β1 itself induces ROS production as part of its signal-transduction pathway^20^. ROS also play an important role in the pERK1/2, phopho-signal transducer and activator of transcription 3 (pSTAT3) and bFGF signaling pathway^21^. These data suggest that ROS induce possibly TGF-β1 and bFGF production. The SHR demonstrated significantly increased oxidative stress levels, as confirmed by the up-regulation of the MDA concentration. The increase in the oxidative stress levels correlates with increase in mild hypoxia, and further induced directly or indirectly the production of TGF-β1 and bFGF. Production of TGF-β1 and bFGF in the prostate leads to stromal proliferation, transdifferentiation and extracellular matrix production.

According to the data by Yono et al., as well as in the present study, the ventral prostate in the SHR is shown in a mild hypoxic condition^9^. The master regulation of oxygen homeostasis is HIF-1, which activates the transcription of hundreds of target genes in response to reduced oxygen availability^22^,^23^,^24^. Berger et al. reported that HIF-1 is activated in a time-dependent manner after exposure of stromal cells to hypoxic conditions, and that secretion of vascular endothelial growth factor (VEGF), FGF-7, TGF-β, FGF-2 (also known as bFGF), and IL-8 is increased under hypoxic *in vitro* conditions in comparison to normoxia in the human prostatic stromal cell culture^25^ Recently, Morelli et al. reported that the SHR prostate is markedly hypoxic and positive for HIF-1α^26^. These data are also in accordance with our findings in this study. Interactions between TGF-β1 and HIFs have been described in a number of experimental systems.

Expression and secretion of TGF-β1 is increased in BPH, and TGF-β1 is produced by basal cells^27^. TGF-β1 is thought to generate a reactive stroma composed of myofibroblasts and fibroblasts that strongly express extracellular matrix components. Prostatic fibroblasts undergo transdifferentiation into myofibroblasts/smooth muscle cells upon stimulation with TGF-β1^28^,^29^,^30^. In addition, bFGF is expressed and secreted by basal epithelial cells of the human prostate^31^, and increased bFGF levels have been observed in early stages of BPH^32^. The corresponding receptor FGF-R1 is present on stromal and basal cells^33^ and stromal cells in culture respond to bFGF by increased proliferation^34^. From current data, it is possible that increases in TGF-β1 and bFGF develop in hyperplasia in the ventral prostate in the SHR.

In the hyperplastic prostate 40% of the cellular volume is composed of smooth muscle^35^. In a study by Kyprianou et al. the authors have found an intense immunoreactivity for α-SMA, which was abundant in stromal smooth muscle in BPH human prostate^36^. In our study we found a significant overexpression of the α-SMA protein levels in the SHR group, which is in accordance with the above study.

In order to confirm our hypothesis, 12-week-old male SHRs were treated with nicorandil for six weeks. Previously, we have investigated the effects of nicorandil in the hypertension-related dysfunction in the bladder of SHRs and we found a significant increase in the bladder blood flow which ameliorated the bladder dysfunction^15^; so we considered that nicorandil could increase the prostatic blood flow, as well. Indeed we have shown that nicorandil significantly increased the blood flow in the prostate of SHRs. Subsequently, increased tissue levels of MDA, HIF-1α, TGF-β1 and bFGF were ameliorated, and the development of hyperplasia in the ventral prostate was inhibited in the nicorandil treated SHR. Recently, nicorandil is reported to reduce ROS production^37^. Serizawa et al. reported that nicorandil protects against paclitaxel-induced endothelial dysfunction, which may be brought about by improved NO bioavailability due to the reduction of oxidative stress via K_ATP_ channel activation in rat femoral artery^37^. Furthermore, Tajima et al. reported the protective effect of nicorandil on the endothelium was mediated by inhibition of NADPH oxidase activity via mitochondrial K_ATP_ channel opening in hypoxia-reoxygenation treated endothelial cells^38^. Another possibility is that nicorandil inhibits TGF-β1 production directly. Sudo et al. reported that nicorandil significantly prevented the overexpression of type I collagen, fibronectin, TGF-β, and platelet-derived growth factor mRNA in anti-Thy1 nephritis rat kidney^39^. Furthermore, as cross talk between ROS production, growth factors and cytokines network have been reported other than prostate, further studies are required to clarify the mechanism to develop in BPH/BPE.

In conclusion, the present study demonstrated that SHRs showed significant increases in blood pressure, tissue levels of MDA, HIF-1α, TGF-β1, bFGF and α-SMA, and a significant decrease in the prostatic blood flow. Although treatment with nicorandil failed to decrease the blood pressure, it ameliorated these factors and inhibited the development of ventral prostatic hyperplasia. We propose that development of prostatic hyperplasia is related to prostatic hypoxia, which nicorandil prevents via its ability to increase the blood flow in the prostate.

## Methods

### Animal preparation

All animal experiments were approved by the Institutional Animal Care and Use Committee of Tottori University (#09-Y-68). Six-week-old male SHRs and WKYs were purchased from SLC (Shizuoka, Japan). In our preliminarily experiments, we confirmed the effective doses of nicorandil (daily treatments for six weeks with 0.3, 1.0, 3 or 10 mg/kg, intraperitoneally (i.p.)) in the SHR evaluating blood flow in the prostate, and we adopted two doses (3 and 10 mg/kg, i.p.) in the present study. In addition daily treatments for six weeks with nicorandil of 3 or 10 mg/kg, i.p. did not alter the prostatic blood flow in the WKY (n = 4). All rats were kept under identical conditions, and had access to food and drinking water *ad libitum*. At the age of 12 weeks, the rats were divided into four groups (each group n = 8); an age-matched WKY group treated with vehicle (saline), i.p. (WKY), SHRs treated with vehicle (saline), i.p. (SHR), and SHRs treated with nicorandil at a daily dose of 3 or 10 mg/kg, i.p. (Nic3 and Nic10, respectively). Upon reaching 18 weeks of age, the blood pressure and heart rate were measured by warming the whole animal body without anesthesia by the tail cuff method (BP-98A-L, Softron, Tokyo, Japan) according to our previous reports^15^,^40^. In short, measurements of blood pressure were performed three times, and the average values were adopted as their individual values. Subsequently, the prostatic blood flow was measured under sodium pentobarbital (50 mg/kg, i.p.), and then, the rats were sacrificed with an overdose of sodium pentobarbital (60 mg/kg i.p.). The doses of nicorandil have been chosen according to our previous publication^15^. Furthermore by performing preliminary experiments we reassured that there was no effect of the vehicle (saline) in the prostatic blood flow. The isolated ventral prostates were frozen at −80°C until measurements of tissue levels of MDA, HIF-1α, TGF-β1, bFGF, DHT, and α-SMA were performed. The rest of ventral and dorsolateral prostate was put in 10% neutral buffered formalin for histopathologic evaluation.

### Measurement of prostatic blood flow in the rats

Prostatic blood flow was measured with the hydrogen clearance method according to our previous reports^15^,^40^. In short, after saturation of the tissue with hydrogen for followed by inhalation of 9% hydrogen gas in air (at 1 litter per minute), the blood flow value (in ml min^−1^ 100 g^−1^ tissue) was calculated from the half-life of the clearance curve obtained^41^. A tissue blood flow meter with an amplifier (PHG-301; Unique Medical Co., Tokyo, Japan) was employed. The hydrogen electrode (80-μm diameter, UHE-201C; Unique Medical Co., Tokyo, Japan) was inserted into the ventral prostate. A rod-type Ag/AgCl reference electrode (UHE-001; Unique Medical Co., Tokyo, Japan) was inserted between the skin and musculature in the abdomen. In each rat, the prostatic blood flow was measured three times in three different parts of the ventral prostate, and an average value of nine measurements was used for each rat prostatic blood flow. In each group, n = 8.

### Measurement of tissue levels of MDA in the prostate

The tissue levels of MDA, a marker of lipid peroxidation, in the prostatic tissue homogenates were identified by using a commercially available kit (NWLSSTM Malondialdehyde Assay, Northwest Life Science Specialties, LLC, Vancouver, WA) according to manufacturer's instruction. The MDA concentrations were normalized by protein contents.

### Measurement of tissue levels of HIF-1α, TGF-β1, bFGF and DHT in the prostate

The tissue levels of HIF-1α (Enzyme-linked immunosorbent assay Kit for Hypoxia Inducible Factor 1 Alpha (HIF1a), USCN Life Science Inc, Wuhan, China), TGF-β1 (Quantikine® mouse/rat/porcine TGF-β1 immunoassay, R&D Systems, Inc., Minneapolis, MN), bFGF (Rat basic Fibroblast Growth Factor (bFGF) ELISA Kit, CUSABIO BIOTECH, Wuhan, China) and dihydrotestosterone (DIHYDROTESTOSTERONE (DHT) ELISA, BioVendor LLC, Candler, NC) in the prostate were measured by the enzyme-linked immunosorbent assay (ELISA) method, and were carried out according to the kit manufacturer's instructions. The HIF-1α, TGF-β1, bFGF and dihydrotestosterone concentrations were normalized by protein contents.

### Determination of α-SMA and β-actin by Western blot analysis

Briefly, prostate samples (50 mg) were homogenized in 250 μl lysis buffer: 1% NP-40, 0.05% sodium deoxycholate, 0.1% SDS, 100 mM NaCl, 50 mM Tris/HCl, pH 7.5, 2.0 mM EDTA with Complete Protease Inhibitor Cocktail tablet (Roche Diagnostics, Indianapolis, IN, USA). The homogenates were subjected to centrifugation at 10,000 × g for 20 min at 4°C. The supernatants were collected and used for colometric detection and quantitation of total protein kit (Protein Assay Rapid Kit, Wako Pure Chemical, Osaka, Japan). The protein samples (50 μg) were subjected to SDS-polyacrylamide gel electrophoresis (10% gradient gels). Proteins were electrophoretically transferred to polyvinylidene difluoride (PVDF) membranes, blocked with Tris-Buffered Saline (TBS), 0.1% Tween 20 (TBS-T) containing 5% nonfat dried milk, washed with TBS-T, and incubated overnight at 4°C with antibodies for α-SMA (1:400) and anti-β-actin (1:5000) in TBS-T containing 5% nonfat dried milk. The blots were washed with TBS-T (6 times × 10 min) and incubated for one hour on ice on a shaker with secondary antibody conjugated with horseradish peroxidase (1:3000) in TBS-T containing 5% nonfat dried milk. After thorough washing with TBS-T (6 times × 10 min) the detection was performed using enhanced chemiluminescence reagent.

The antibodies used were as follows: rabbit polyclonal antibody which detects the anti-α-SMA (ab5694 Abcam, Tokyo, Japan), and rabbit polyclonal antibody for anti-β-actin (Catalog No: 54590, AnaSpec, Inc., San Jose, CA). Anti-β-actin was used as a loading control for normalization.

### Histological examinations in the rat prostate

After fixation, the tissues were embedded in paraffin, and five-micron-thick tissue sections were cut from the paraffin blocks. All of the prostate specimens were stained using hematoxylin and eosin (H & E). Each section was viewed under a light microscope at a magnification of ×40–400.

### Determination of the protein concentration in the prostate

The protein concentration was determined using a commercial kit (Protein Assay Rapid Kit, Wako Pure Chemical, Osaka, Japan).

### Data analysis

Data are shown as means ± S.E.M. of eight separate determinations in each group. A statistical comparison of differences among the groups was performed using analysis of variance and Fisher's multiple comparison tests. P values less than 0.05 were regarded as the level of significance.

### Drugs and chemicals

Nicorandil was kindly supplied by Chugai Pharmaceutical Co. Ltd (Tokyo, Japan). All other chemicals were commercially available and of reagent grade.

## Author Contributions

M.S. designed experiments and wrote the main manuscript text. M.S., R.O., P.T. and S.S. performed experiments. M.S., P.T. and R.O. analyzed the data. Y.K. prepared the figures. M.H., T.S. and S.T. conducted the critical revision of the manuscript for important intellectual content. All authors reviewed the manuscript.

## Figures and Tables

**Table 1:** General Features of the experimental rats

	Body Weight	Prostate Weight	PBR	Heart rate	Blood pressure (mmHg)		
Group	(g)	(mg)	(×10^−3^)	(/min)	Systolic	Mean	Diastolic
WKY (n = 8)	400 ± 4	662 ± 34	1.66 ± 0.09	326 ± 11	128.1 ± 1.2	107.4 ± 2.7	96.1 ± 1.1
SHR (n = 8)	305 ± 6*	581 ± 34	1.99 ± 0.06*	329 ± 22	198.0 ± 3.7*	171.8 ± 2.7*	146.3 ± 12.9*
Nic3 (n = 8)	306 ± 6*	617 ± 12	2.03 ± 0.04*	336 ± 10	191.3 ± 5.1*	163.3 ± 4.4*	144.3 ± 6.3*
Nic10 (n = 8)	282 ± 4*#	541 ± 24*§	1.93 ± 0.13	300 ± 10	188.3 ± 4.5*	161.3 ± 4.4*	144.8 ± 3.4*

PBR: Prostate Body weight Ratio. WKY: 18-week-old Wistar-Kyoto rat group; SHR: 18-week-old SHR group; Nic3: 18-week-old SHRs treated with nicorandil at a daily dose of 3 mg/kg, i.p.; Nic10: 18-week-old SHRs treated with nicorandil at a daily dose of 10 mg/kg, i.p.

Data are shown as means ± S.E.M. of eight separate determinations in each group. *: Significantly different from the WKY group (P < 0.05 is level of significance); #: Significantly different from the SHR (P < 0.05 is level of significance); ^§^: Significantly different from the Nic3 (P < 0.05 is level of significance).
